# Analyzing brain structural differences associated with categories of blood pressure in adults using empirical kernel mapping-based kernel ELM+

**DOI:** 10.1186/s12938-019-0740-4

**Published:** 2019-12-27

**Authors:** Xinying Yu, Bo Peng, Zeyu Xue, Hamidreza Saligheh Rad, Zhenlin Cai, Jun Shi, Jianbing Zhu, Yakang Dai

**Affiliations:** 1 Shanghai Institute for Advanced Communication and Data Science, School of Communication and Information Engineering, Shanghai University, Shanghai, China; 2 Suzhou Institute of Biomedical Engineering and Technology, Chinese Academy of Science, Suzhou, Jiangsu China; 3 Quantitative Medical Imaging Systems Group, Research Center for Molecular and Cellular Imaging, Institute for Advanced Medical Technologies and Devices, Tehran University of Medical Sciences, Tehran, Iran; 4 The Affiliated Suzhou Science & Technology Town Hospital of Nanjing Medical University, Suzhou, Jiangsu China; 5 Suzhou Science & Technology Town Hospital, Suzhou, 215153 Jiangsu China; 6 Suzhou Key Laboratory of Medical and Health Information Technology, Suzhou, China; 7 Nanjing Guoke Medical Engineering Technology Development Co., Ltd, Nanjing, China; 8 Jinan Guoke Medical Engineering Technology Development Co., Ltd, Jinan, China

**Keywords:** Hypertension, Magnetic resonance imaging (MRI), Kernel extreme learning machine plus (KELM+), Empirical kernel mapping (EKM), Regions of interest (ROI) features, Brain network features

## Abstract

**Background:**

Hypertension increases the risk of angiocardiopathy and cognitive disorder. Blood pressure has four categories: normal, elevated, hypertension stage 1 and hypertension stage 2. The quantitative analysis of hypertension helps determine disease status, prognosis assessment, guidance and management, but is not well studied in the framework of machine learning.

**Methods:**

We proposed empirical kernel mapping-based kernel extreme learning machine plus (EKM–KELM+) classifier to discriminate different blood pressure grades in adults from structural brain MR images. ELM+ is the extended version of ELM, which integrates the additional privileged information about training samples in ELM to help train a more effective classifier. In this work, we extracted gray matter volume (GMV), white matter volume, cerebrospinal fluid volume, cortical surface area, cortical thickness from structural brain MR images, and constructed brain network features based on thickness. After feature selection and EKM, the enhanced features are obtained. Then, we select one feature type as the main feature to feed into KELM+, and the rest of the feature types are PI to assist the main feature to train 5 KELM+ classifiers. Finally, the 5 KELM+ classifiers are ensemble to predict classification result in the test stage, while PI is not used during testing.

**Results:**

We evaluated the performance of the proposed EKM–KELM+ method using four grades of hypertension data (73 samples for each grade). The experimental results show that the GMV performs observably better than any other feature types with a comparatively higher classification accuracy of 77.37% (Grade 1 vs. Grade 2), 93.19% (Grade 1 vs. Grade 3), and 95.15% (Grade 1 vs. Grade 4). The most discriminative brain regions found using our method are olfactory, orbitofrontal cortex (inferior), supplementary motor area, etc.

**Conclusions:**

Using region of interest features and brain network features, EKM–KELM+ is proposed to study the most discriminative regions that have obvious structural changes in different blood pressure grades. The discriminative features that are selected using our method are consistent with the existing neuroimaging studies. Moreover, our study provides a potential approach to take effective interventions in the early period, when the blood pressure makes minor impacts on the brain structure and function.

## Background

Hypertension is one of the risk factors for cognitive dysfunction. According to the epidemiological survey, the global incidence of hypertension in 2000 was about 26.4%, affecting 972 million people worldwide. By 2025, the number of people affected by hypertension is to increase by 60% to 1.56 billion [1]. A long-term follow-up of elderly patients at risk for cardiovascular disease found that the patient’s blood pressure (BP) variability affects the patient’s cognitive function [2]. A latitudinal investigation demonstrates that high systolic blood pressure (SBP), high diastolic blood pressure (DBP) and persistent hypertension can accelerate the decline of cognitive function, as well as increase the incidence of dementia [3]. Longitudinal studies have found that antihypertensive therapy can effectively reduce the incidence of cognitive dysfunction [4]. Excessive BP can cause cerebral vascular damage, which in turn causes white matter and gray matter ischemic or hemorrhagic damage [5], while white matter and gray matter ischemia can cause brain atrophy and leukoaraiosis. All these studies indicate that high BP may affect cognitive function.

Hypertension can be classified by severity. The classification scheme for hypertension helps determine the condition, quantify the risk, evaluate the prognosis and guide the management [6]. The “2017 American College of Cardiology/American Heart Association (2017 ACC/AHA) Guideline for the Prevention, Detection, Evaluation, and Management of High Blood Pressure in Adults” recently recommended a new categorization for BP grades. This new guideline commends that BP should be classified in four categories: normal (Grade 1), elevated (Grade 2), hypertension stage 1 (Grade 3) and 2 (Grade 4). And defined hypertension as a SBP of ≥ 130 mmHg and/or a DBP of ≥ 80 mmHg, reducing the former SBP and DBP by 10 mmHg (a SBP of ≥ 140 mmHg and/or DBP of ≥ 90 mmHg [7]). The research of Ettehad [8] and Xie et al. [9] also supported this BP ≥ 130/80 mmHg as critical value of hypertension intervention.

The overall situation of prevention and control of hypertension in China is severe. At present, Chinese diagnostic criteria of hypertension is still BP ≥ 140/90 mmHg. According to the 2017 ACC/AHA new diagnostic criteria of hypertension, China will add another 100 million hypertensive patients. Treatment in the early stages of disease development may help prevent the development of cardiovascular disease and reduce the risk and complications of hypertension [10, 11]. It is necessary for us to learn from the 2017 ACC/AHA guidelines, which is of great significance for the prevention and control of hypertension as well as the entire chronic patient population in China.

The purpose of this study is using machine learning to explore the relationship between BP grades and brain structural changes. Magnetic resonance (MR) imaging, a safe and effective means, plays an important role in revealing brain abnormalities. ROI-based analysis has been widely used [12]. Maaike et al. [13] used voxel-based morphometry to study the gray matter and white matter volume of hypertension, revealing the relationship between hypertension and anterior cingulate cortex (ACC), lower forehead (IFG) and hippocampal volume. Studies of structural abnormalities in the brain based on MR images of hypertensive patients have shown that brain atrophy and brain tissue lesions often occurred in gray matter and white matter [14, 15], affecting the transport of nutrients to neurons and leading to the decline of cognitive function [16]. From MR-related studies, it is known that gray matter damages appeared in the prefrontal cortex, hippocampus, lower jaw, and inferior parietal lobe, white matter lesions mainly occurs in the frontal area [17, 18]. Peter et al. [19] demonstrated that atrophy of the auxiliary motor areas, superior frontal gyrus, anterior cingulate cortex and middle temporal lobe is associated with hypertension. In addition, high BP gives rise to atrophy of the medial temporal lobe, which plays an important role in cognitive development [20]. Detection of hypertension-related brain regions is of great value in clinical and academic studies. Those researches above have only studied hypertension brain morphometry. Their subjects consist of normal group and hypertension group whose diagnostic criterion is BP ≥ 140/90 mmHg. And less use automated classification to extract hypertension-related brain regions. Therefore, more studies are needed to further explain the relationship between BP grades and brain morphometry.

In this paper, we examined the hypertension-related brain morphometry in regions of interest (ROIs) using features, which consist of ROI features and brain network features. ROI features were extracted from the brain structural MR images including gray matter volume (GMV), white matter volume (WMV), cerebrospinal fluid volume (CSFV), cortical thickness (Thickness), and cortical surface area (Area). Brain network features were constructed by computing the correlation index of cortical thickness values between ROIs. The two feature types complement each other in revealing neuroanatomical information about hypertension.

Due to the complexity of brain diseases, the use of single information cannot fully represent the disease characteristics in process of the diagnosis. For this reason, comprehensive consideration of multiple information is required. Learning Using Privileged Information (LUPI), a new learning paradigm for classifier proposed by Vapnik and Vashist, can be a good way to solve this problem. The privileged information (PI) is only available during the training phase of model, but unavailable during the testing phase [21]. PI can help establish better prediction rules by providing additional information to training samples. It has become a trend for researchers to embed LUPI paradigm in different classifiers, such as the support vector machine plus (SVM+) and random vector functional link network plus (RVFL+) [22], which usually achieves improved classification performance [21].

The proposed kernel-based ELM+ (KELM+) is developed based on kernel-based RVFL+ (KRVFL+) [22]. ELM and RVFL, two kinds of classifiers based on single-layer feed-forward neural network (SLFN) [23], have received extensive attention in recent years. With high approximation ability, good generalization performance and very fast training time, ELM is widely used for a variety of classification tasks [24]. However, random affine transformation in ELM+ usually causes prediction instability. To this end, we propose a KELM+ algorithm to overcome this problem and improve performance. KRVFL+ outperforms SVM+ on several benchmark datasets [22]. In view of the nuances of ELM and RVFL, we also consider that KELM+ outperforms SVM+ in the network structure.

Empirical kernel mapping (EKM), one of the kernel methods, can map raw data to a high-dimensional data space via the inner-product forms [25], which works as the implicit kernel mapping (IKM) [25]. EKM overcomes the limitations of traditional IKM on inner-product calculation, and can explicitly map samples to feature space. In the meanwhile, it can fully retain the structural characteristics of data [26].

In this study, we proposed an EKM-based KELM+ (EKM–KELM+) method, which can be used to investigate brain structural differences in different grades of BP. Specifically, first EKM performed on six types of feature to generate six enhanced features. Then, one type of feature is selected as the main feature, and the other five features are used as PI, together with the main feature to form five feature pairs, which are built to train five individual KELM+ classifiers. Finally, ensemble learning is performed on the KELM+ classifiers to give the classification result.

The main contributions of the method are twofold: (1) by transforming the original features to high-dimensional to form enhancement features through EKM, EKM–KELM+ has a more meaningful input layer in the neural network, which help improving classification performance; (2) instead of using simple multi-level ROI for mixed feature selection, one soft tissue feature is selected as main feature, and the other five features are used as PI to assist the classifiers training. Only the main feature is used in the testing. The most discriminative brain regions, which have structural changes affected by hypertension, can be found using our method. This can also help us to analyze the changes of specific brain regions in BP from grade 2 to grade 4. Moreover, our study provides a potential approach to take effective interventions in the early period, when the BP has minor impacts on the brain structure and function.

## Results

The proposed EKM–KELM+ algorithm is compared with the following algorithms: (1) SVM classifier with Radial Basis Function (RBF) kernel is used for every ROI feature; (2) KELM classifier is used for every ROI feature; (3) KELM+ without EKM.

In this experiment, the fivefold cross-validation (CV) strategy was conducted; for each round of CV, the performance of the model can be calculated separately, which reduces the variance of the evaluation. The classification accuracy (ACC), sensitivity (SEN), specificity (SPC), Youden index (YI), positive predictive value (PPV), negative predictive value (NPV) and F1-score (F1) are used as evaluation indices. Our classification results were presented in the form of mean ± SD.

### Classification performance

Table 1 gives the classification performance using different feature types between Grade 1 and Grade 2, Grade 1 and Grade 3 and Grade 1 and Grade 4. For Grade 1 and Grade 2; in the comparison of different feature types, the cortical thickness performs worst in all feature types. It is found that the GMV performs observably better than any other volumetric features (i.e., WMV and CSFV) with a comparatively higher classification accuracy of 76.73%, sensitivity of 78.73%, and specificity of 75.14%. Similarly, cortical thickness performs worst and GMV performs best with an accuracy of 93.19%, sensitivity of 93.14%, and specificity of 93.23% in Grade 1 and Grade 3. In Grade 1 and Grade 4 group, GMV has the highest classification accuracy of 95.15%, sensitivity of 97.14%, and specificity of 93.14%, while WMV performs worst.

**Table 1:** Classification performance using different feature types between Grade 1 and Grade 2, Grade 1 and Grade 3 and Grade 1 and Grade 4 (mean ± std, UNIT: %)

	GMV	WMV	CSFV	Thickness	Area
*Grade 1 and Grade 2*					
ACC	76.73 ± 4.39	73.20 ± 5.13	76.63 ± 6.04	70.52 ± 4.84	75.98 ± 2.18
SEN	78.73 ± 6.43	75.97 ± 6.99	79.56 ± 12.17	58.21 ± 21.23	77.19 ± 5.53
SPC	75.14 ± 13.01	70.75 ± 12.48	73.19 ± 13.99	81.75 ± 20.80	75.03 ± 4.17
PPV	75.58 ± 9.33	71.41 ± 6.66	75.08 ± 9.47	80.88 ± 14.18	74.10 ± 2.02
NPV	79.59 ± 3.29	76.04 ± 6.25	81.33 ± 11.09	69.74 ± 7.51	78.24 ± 4.64
YI	53.88 ± 7.85	46.72 ± 10.51	52.75 ± 11.40	39.96 ± 8.63	52.23 ± 4.55
F1	76.52 ± 3.06	73.20 ± 3.07	76.49 ± 5.49	63.82 ± 11.27	75.45 ± 2.19
*Grade 1 and Grade 3*					
ACC	93.19 ± 4.01	83.70 ± 6.97	80.87 ± 5.97	80.05 ± 5.56	83.69 ± 8.50
SEN	93.14 ± 0.26	78.38 ± 10.62	86.38 ± 8.47	76.76 ± 5.97	83.71 ± 9.89
SPC	93.23 ± 8.16	89.24 ± 10.06	75.33 ± 7.63	83.42 ± 6.72	83.62 ± 7.92
PPV	93.70 ± 7.22	88.64 ± 9.82	77.92 ± 6.37	82.41 ± 6.16	83.66 ± 7.47
NPV	93.11 ± 0.55	80.86 ± 8.59	85.35 ± 9.13	78.16 ± 5.92	83.99 ± 8.97
YI	86.38 ± 8.07	67.62 ± 14.06	61.71 ± 11.87	60.19 ± 11.09	67.33 ± 16.07
F1	93.31 ± 3.65	82.68 ± 7.27	81.80 ± 5.95	79.41 ± 5.38	83.61 ± 8.81
*Grade 1 and Grade 4*					
ACC	95.15 ± 3.98	82.93 ± 4.56	88.24 ± 5.50	86.91 ± 5.43	84.27 ± 3.14
SEN	97.14 ± 3.91	80.76 ± 7.69	88.95 ± 6.45	86.19 ± 5.23	83.52 ± 3.61
SPC	93.14 ± 4.72	85.04 ± 7.13	87.52 ± 6.15	87.71 ± 7.71	85.05 ± 5.53
PPV	93.40 ± 4.73	84.59 ± 5.85	87.84 ± 5.37	87.69 ± 7.74	85.15 ± 4.62
NPV	97.14 ± 3.91	81.98 ± 5.08	88.83 ± 6.17	86.45 ± 5.10	83.84 ± 3.71
YI	90.28 ± 7.85	65.81 ± 9.31	76.47 ± 11.08	73.90 ± 10.99	68.57 ± 6.24
F1	95.21 ± 4.00	82.42 ± 5.23	88.33 ± 5.44	86.83 ± 5.43	84.17 ± 2.36

*GMV* gray matter volume, *WMV* white matter volume, *CSFV* cerebrospinal volume, thickness, cortical thickness, *Area* cortical surface area, *ACC* accuracy, *SEN* sensitivity, *SPC* specificity, *PPV* positive predictive value, *NPV* negative predictive value, *YI* Youden’s index, *F1* F1-score

It can be seen from Table 1 that all the best results are achieved on GMV. It means that the high BP group and the normal BP group have more differences in GMV than in others. On every type of feature, the classification accuracy increases with the increase of BP grade, which indicates that higher BP will aggravate the change of ROI feature.

Table 2 gives the classification results of different algorithms on the different feature types. It can be found that the proposed EKM–KELM+ outperforms all the compared algorithms.

**Table 2:** Comparison with different types of features using different algorithms on classification accuracy (mean ± std, UNIT: %)

	GMV	WMV	CSFV	Thickness	Area
*Grade 1 and Grade 2*					
SVM	60.90 ± 7.21	58.21 ± 5.56	58.90 ± 9.67	54.09 ± 8.96	54.81 ± 8.52
KELM	70.47 ± 6.11	66.40 ± 4.11	67.75 ± 4.95	68.49 ± 4.32	70.49 ± 3.58
KELM+	74.34 ± 5.40	69.85 ± 4.57	73.89 ± 5.52	73.32 ± 9.42	69.85 ± 6.63
EKM–KELM+	76.73 ± 4.39	73.20 ± 5.13	76.63 ± 6.04	70.52 ± 4.84	75.98 ± 2.18
*Grade 1 and Grade 3*					
SVM	78.13 ± 6.41	66.47 ± 5.27	61.11 ± 10.89	67.70 ± 8.81	69.27 ± 9.69
KELM	82.24 ± 7.19	72.70 ± 7.42	69.87 ± 4.88	77.99 ± 7.15	74.77 ± 11.24
KELM+	89.05 ± 4.40	80.29 ± 7.28	77.46 ± 4.74	78.70 ± 5.97	83.67 ± 8.10
EKM–KELM+	93.19 ± 4.01	83.70 ± 6.97	80.87 ± 5.97	80.05 ± 5.56	83.69 ± 8.50
*Grade 1 and Grade 4*					
SVM	87.65 ± 3.93	72.63 ± 5.72	76.61 ± 5.04	78.61 ± 8.03	71.92 ± 3.56
KELM	88.98 ± 6.20	80.82 ± 7.91	83.48 ± 3.37	80.75 ± 5.52	84.20 ± 5.87
KELM+	92.43 ± 3.00	82.25 ± 5.42	86.22 ± 3.78	86.91 ± 5.43	84.22 ± 3.92
EKM–KELM+	95.15 ± 3.98	82.93 ± 4.56	88.24 ± 5.50	86.91 ± 5.43	84.27 ± 3.14

### Experiment on kernel type

**Fig. 1:**
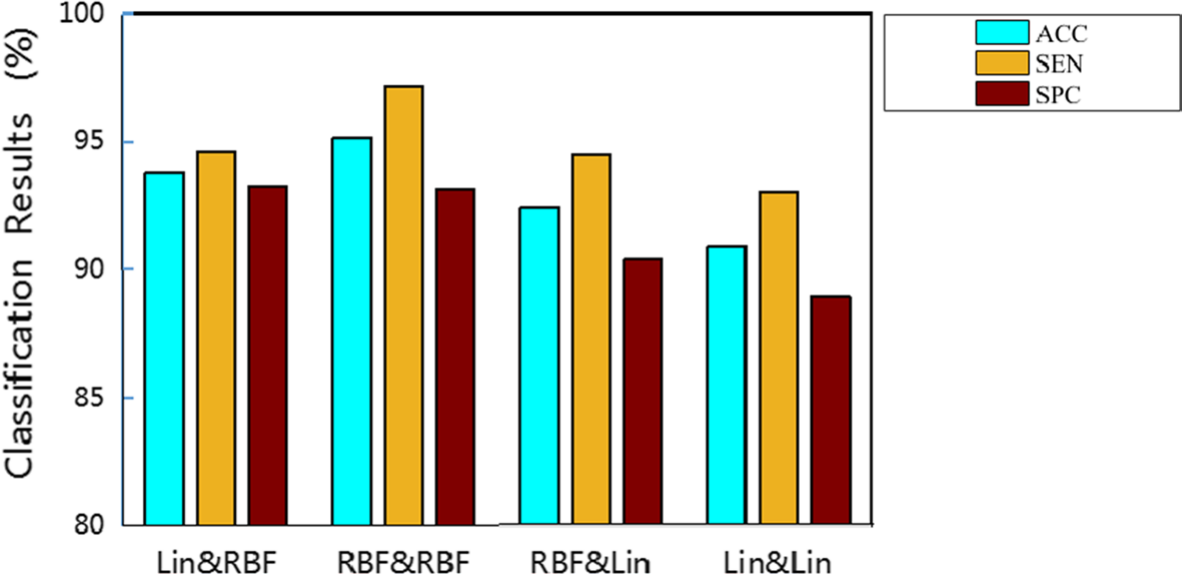
Classification results of Grade 1 vs. Grade 4, using EKM–KELM+ with different kernel types (EKM and KELM+) on the GMV feature

Different kernel function types represent different ways of data mapping. Polynomial kernel, RBF kernel, and linear kernel are mostly used kernel types. In this study, we used RBF kernel and linear kernel. We chose the most suitable kernel function type through experiments to achieve the best classification performance. Classification results of Grade 1 vs. Grade 4, using EKM–KELM+ with different kernel types (RBF kernel or linear kernel of EKM & KELM+) on the GMV feature are shown in Fig. 1. Experimental results show that the kernel function has an important impact on the performance of the classification. Using RBF kernel for EKM and KELM+ can achieve the best classification performance, which reflects the robustness of our method. The RBF kernel function is commonly used as the kernel functions for the reason that is has good anti-interference ability for noise in the data.

### The most discriminative features

The most discriminative features are selected from ROI features and brain network features, respectively. The top 10 of the most discriminative ROI features and brain network features for Grade 2, Grade 3 and Grade 4 compared with Grade 1 are listed in Table 3.

**Table 3:** Top 10 of the most discriminative ROI features and correlative features that were selected using the proposed classification framework

No.	ROI features	Frequency	Correlative features	Frequency
*Grade 1 and Grade 2*				
1	Orbitofrontal cortex (superior)_R	25	Inferior frontal gyrus (opercular)_L-inferior frontal gyrus (opercular)_R	5
2	Superior temporal gyrus_R	25	Inferior frontal gyrus (opercular)_L-insula_R	5
3	Middle temporal gyrus_L	23	Inferior frontal gyrus (opercular)_L-anterior cingulate gyrus_R	5
4	Angular gyrus_L	22	Inferior frontal gyrus (opercular)_L-precuneus_R	5
5	Precuneus_R	22	Superior parietal gyrus_L-precuneus_R	5
6	Superior temporal gyrus_L	22	Inferior frontal gyrus (opercular)_L-caudate_L	5
7	Supramarginal gyrus_L	21	Posterior cingulate gyrus_L-pallidum_R	5
8	Angular gyrus_R	21	Orbitofrontal cortex (superior)_L-inferior frontal gyrus (opercular)_L	4
9	Temporal pole (superior)_R	21	Inferior frontal gyrus (opercular)_L-inferior frontal gyrus (triangular)_L	4
10	Inferior frontal gyrus (opercular)_R	20	Inferior frontal gyrus (opercular)_L-anterior cingulate gyrus_L	4
*Grade 1 and Grade 3*				
1	Rolandic operculum_R	25	Superior frontal gyrus (medial) _R-posterior cingulate gyrus_L	5
2	Rectus gyrus_R	24	Olfactory_L-parahippocampal gyrus_R	5
3	Insula_R	24	Rolandic operculum_L-cuneus_L	5
4	Superior-temporal gyrus_L	24	Olfactory_L-superior occipital gyrus _L	5
5	Superior frontal gyrus (dorsal) _L	23	Superior frontal gyrus (medial) _L-superior occipital gyrus _L	5
6	Orbitofrontal cortex (superior) _L	23	Cuneus_L-fusiform gyrus_R	5
7	Superior temporal gyrus _R	23	ParaHippocampal gyrus_R-superior parietal gyrus _R	5
8	Inferior temporal gyrus _L	23	Posterior cingulate gyrus_L-supramarginal gyrus _L	5
9	Orbitofrontal cortex (medial) _R	22	Superior occipital gyrus_L-supramarginal gyrus _L	5
10	Middle temporal gyrus _R	21	Superior occipital gyrus_L-precuneus_R	5
*Grade 1 and Grade 4*				
1	Superior temporal gyrus_L	25	Inferior frontal gyrus (opercular) _R-middle cingulate gyrus _R	5
2	Superior frontal gyrus (dorsal) _L	23	Orbitofrontal cortex (medial) _R-posterior cingulate gyrus_L	5
3	Orbitofrontal cortex (superior) _R	23	Middle cingulate gyrus_R-middle occipital gyrus _L	5
4	Inferior frontal gyrus (triangular) _L	22	Posterior cingulate gyrus_L-angular gyrus_L	5
5	Supplementary motor area_L	22	Middle cingulate gyrus_L-paracentral lobule _R	5
6	Supplementary motor area_R	22	Inferior frontal gyrus (opercular) _R-putamen_L	5
7	Rectus gyrus_R	22	Superior frontal gyrus (medial) _R-putamen_L	5
8	Superior temporal gyrus_R	22	Orbitofrontal cortex (medial) _L-putamen_L	5
9	Middle frontal gyrus_L	21	Hippocampus_L-putamen_L	5
10	Orbitofrontal cortex (inferior) _L	21	ParaHippocampal gyrus_L-putamen_L	5

*R* right hemisphere, *L* left hemisphere

For Grade 2 compared with Grade 1, the top 10 of the most discriminative ROI features are mainly distributed in frontal lobe [inferior frontal gyrus (opercular) right, olfactory right], temporal lobe (bilateral superior temporal gyrus, middle temporal gyrus left), limbic lobe (temporal pole (superior) right), and parietal lobe (bilateral angular gyrus, precuneus right, supramarginal gyrus left).

For Grade 3, the main distribution of the top 10 discriminative ROI features is in frontal lobe (rectus gyrus right, superior frontal gyrus (dorsal) left, orbitofrontal cortex (superior) left, orbitofrontal cortex (medial) right), temporal lobe (bilateral superior temporal gyrus, bilateral Inferior temporal gyrus, bilateral middle temporal gyrus), bilateral Insula, and central region (rolandic operculum right), which compared with Grade 1.

As for Grade 4, the top 10 of the most discriminative ROI features are found in frontal lobe (superior frontal gyrus (dorsal) left, bilateral orbitofrontal cortex (superior), bilateral orbitofrontal cortex (inferior), bilateral supplementary motor area, inferior frontal gyrus (triangular) left, bilateral middle frontal gyrus, rectus gyrus right), and temporal lobe (bilateral superior temporal gyrus).

**Fig. 2:**
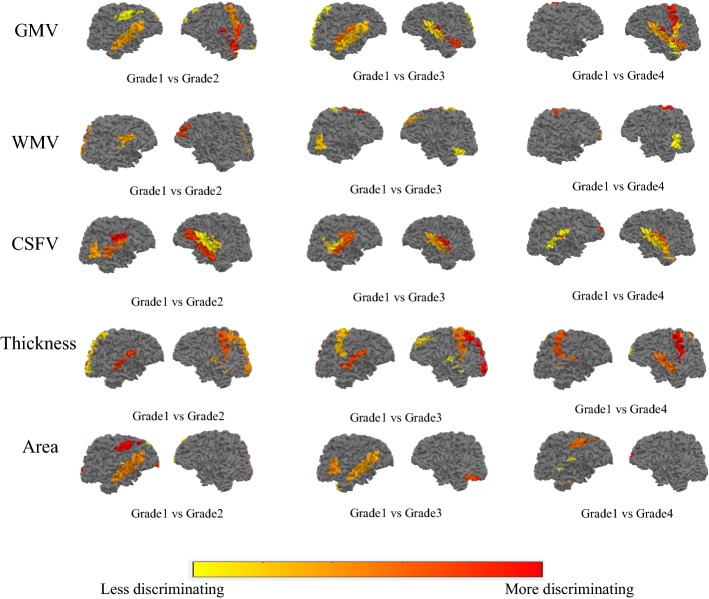
The ROIs with statistically significant decline on volume (GMV, WMV, CSFV), cortical thickness, and surface area are shown. The GMV, WMV, CSFV, thickness, and area were encoded by the color from yellow (small, thin) to red (large, thick) (for interpretation of the references to color in this figure legend, the reader is referred to the web version of this article.)

**Fig. 3:**
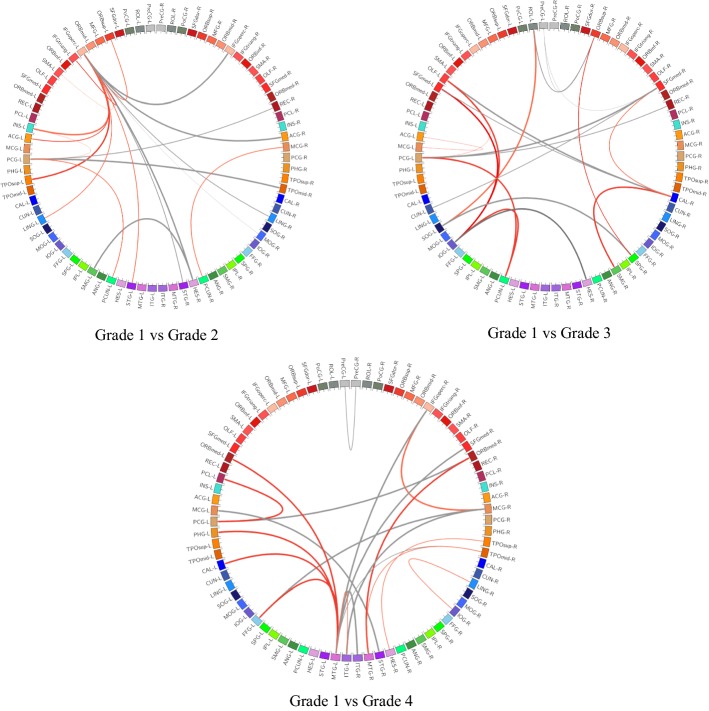
Connection graphs of the most discriminative brain network features (top 20-correlated features) for three groups. Red color lines indicate relation in the same hemisphere, and gray color lines indicate relation in the two sides of the brain. Thickness of each line reflects its selection frequency, e.g., a thicker line indicates a higher selection frequency

Figure 2 shows the results of projecting the most discriminative ROI features (top-10) onto the cortical surface. Three connection graphs of the most discriminative brain network features for three groups are shown in Fig. 3 (top-20), which are generated by Circos software [27]. Thicker line in the connection graph indicates stronger connection between ROIs, while thinner line implies weaker connection. The red lines represent brain connections in the same hemisphere, while the gray lines represent brain connections in different hemispheres of the brain. As we can see in lower grade of BP, the most discriminative brain network features are mainly distributed in left hemisphere. As the BP increases, the features will be gradually distributed in the right hemisphere and finally across both the right and left sides of the brain and almost across all brain regions, including frontal lobe, occipital lobe, limbic lobe, parietal lobe, sub-cortical gray nuclei, and central region. Moreover, regions in the bilateral frontal lobes and limbic lobes show close internal relation. That is, the most sensitive biomarkers of hypertension are mainly distributed in frontal lobe and limbic region.

## Discussion

In this work, the proposed EKM–KELM+ algorithm can help study the brain structural differences associated with BP grades and achieve effective classification results. Its effectiveness is demonstrated on datasets of different BP grades.

### Improvement of the proposed method

Due to the complexity of brain diseases, the use of multiple anatomical MRI measures can provide more information to help research the disease. Although the proposed EKM–KELM+ algorithm is based on the LUPI paradigm that required additional modality for PI in previous work, we successfully performed EKM–KELM+ on multi-parameter information of single-modality neuroimaging data in this work. In fact, GMV, WMV, CSFV, thickness and area are extracted from structural brain MRI, brain network features are computed based on cortical thickness between ROIs. During the training phase, the five feature pairs are built to train five individual KELM+ models. While in testing phase, only one type of feature, extracting from structural brain MR images, will be directly fed to the well-trained KELM+ models to give the final classification result, which is flexible and convenient. The use of EKM before KELM+ results in data obtaining a more powerful expression, which improves the classification performance.

A well-classified performance and discriminative features reported in our study are important in clinical studies. By using our model, we can classify hypertension patients as with and without structural brain changes. Clinicians can give the targeted recommendations for initiation of treatment for these two types of patients. It conforms more with the principles of hypertension treatment.

The current studies on hypertension are all in the population with SBP ≥ 140 mmHg or DBP ≥ 90 mmHg (Grade 4), to find specific brain regions related to hypertension. However, these studies have some shortcomings. They only explain the relationship between hypertension and the relevant brain regions in a general way, which has not considered the network activity of specific brain regions. We have fixed the deficiency of these existing methods by using quantitative analysis. This can provide information of both isolated ROI and brain connectivity between pairs ROIs, and help us understand the change pattern of brain morphological in different BP grades.

### Analysis of discriminative ROIs

We performed *t* test between different groups and counted the number of ROIs with significant changes (*p* value < 0.05) of each feature type. Figure 2 shows the results of projecting the most discriminative ROI features (top 10) onto the volumetric and cortical. The GMV, cortical thickness, and surface area encoded by the color from yellow (larger, thicker) to red (smaller, thinner).

For all groups, the most discriminative ROI features include GMV, WMV, CSFV, Thickness, and Area. The most conspicuous regions of GMV reduction are found in frontal lobe, limbic lobe, temporal lobe, parietal lobe, central region, and occipital lobe. The most obvious regions of WMV reduction are in frontal lobe, parietal lobe, occipital lobe, sub-cortical gray nuclei, and limbic lobe. The most evident regions of Thickness volume reduction are frontal lobe, occipital lobe, limbic lobe, parietal lobe, and temporal lobe. The higher the BP, the more reduction of brain tissue occurred. In insula and sub-cortical gray nuclei, the CSFV has positive correlation with the increase of BP. All critical regions are known to be strongly involved in the pathophysiological mechanisms of hypertension.

### Comparison with other methods

Studies have shown that high SBP, high DBP and persistent high BP will lead to cognitive impairment [28]. Morphological studies have shown that different cognitive dysfunction manifestations (such as overall cognitive function, executive ability, memory impairment) are associated with structural changes in specific brain regions. Researchers [29] found that hypertension patients showed atrophy of the prefrontal and hippocampus, while the prefrontal cortex was closely related to executive ability, emotional processing ability, and social cognition. Blood flow in the posterior parietal region of hypertensive patients increased less than that of non-hypertensive patients when they completed the memory task, which indicates that hypertension may damage cognitive function by reducing local cerebral blood flow [30]. Elevated BP is associated with more executive function impairment than memory, which shows a significant decrease compared with the executive function of the non-hypertensive group [31]. Functional magnetic resonance imaging (fMRI) and diffusion tensor imaging (DTI) on 1007 elderly populations (including 405 hypertensive patients) are used to find that impaired executive function and decreased attention caused by hypertension may be associated with decreased white matter integrity and decreased functional connectivity of the frontotemporal lobe. In addition, cortical gray matter atrophy is closely related to executive dysfunction [32]. Hypertension can also cause atrophy of the medial temporal lobe, which plays an important role in cognitive formation [20].

Since there have been few reports on the automatic classification of hypertension grades, we only compared the brain regions that are differentiated in our results with existing hypertension-related morphological studies. Our results also examined the frontal lobe (bilateral orbitofrontal cortex (superior), superior frontal gyrus (dorsal) left, rectus gyrus right), temporal lobe (bilateral superior temporal gyrus, middle temporal gyrus left), central region (rolandic operculum right), insula right, limbic lobe (hippocampus), sub-cortical gray nuclei (thalamus), and parietal lobe (precuneus right) associated with elevated BP. It is consistent with current morphological studies, demonstrating the effectiveness of our classification method in revealing hypertension-related brains. Meanwhile, the central region and insula, which have not been reported in previous hypertension-related studies, were found in our study. Further research is needed to rule out false positives in our results. It can be found that the discriminative ROIs are mostly located in frontal lobe, which is mainly responsible for planning, sequencing and organizing attention, moral judgment and self-control behaviors. This is consistent with the fact that high blood pressure can cause cognitive damages.

### Limitations

Despite the excellent classification performance, our method still has some limitations. First, as a pilot study, we use a relatively small amount of data during machine learning. Second, since our study is based on a universality, the age of subjects is not limited to a specific range. We can take these elements into consideration for further improving the experiment in the future research.

## Conclusion

In summary, the proposed Empirical Kernel Mapping-Based Kernel ELM+ framework can be used in studying the changes of brain structure associated with blood pressure by a quantitative way. One type of feature is used as the main feature, and other different feature types are used as PI. Finally, the result is obtained by ensemble learning. Compared with other algorithms, our method has the best classification accuracy, which can provide more accurate early intervention identification methods and potential guiding significance for the treatment of hypertension patients. The ROI features and the brain network features can be used to locate specific brain regions that process hypertension. The discriminative features selection by EKM–KELM+ is consistent with existing structural studies. Moreover, our study provides an important step in investigating brain structure and brain connective changes associated with hypertension, which offers a potential research direction to further study the mechanisms basis of the cognitive neuroscience of hypertension.

## Materials and methods

### Participants

The structural MRI data utilized in this study were obtained from the Suzhou Science and Technology town hospital that consist 292 adults, aged from 25 to 76 years. The study is approved by the Ethics Committee of the Third Affiliated Hospital of Soochow University. According to the “2017 American College of Cardiology/American Heart Association (2017 ACC/AHA) Guideline for the Prevention, Detection, Evaluation, and Management of High Blood Pressure in Adults”, we classified the data as four grades: Grade 1, Grade 2, Grade 3, and Grade 4 (more details in Table 4). Each grade includes 73 subjects. Each participant received a structured clinical interview by a psychiatrist to rule out smoking, secondary hypertension, traumatic head injury, diabetes, and congestive heart failure or pulmonary disease. Characteristics of all subjects are shown in Table 5.

**Table 4:** Four grades according to 2017 ACC/AHA

Grade	BP category	SBP (mmHg)		DBP (mmHg)
Grade 1	Normal	< 120	and	< 80
Grade 2	Elevated	120–129	and	< 80
Grade 3	Hypertension stage 1	130–139	and/or	80–89
Grade 4	Hypertension stage 2	> 140	and/or	≥ 90
	Hypertension crisis	> 180	and/or	> 120

*BP* blood pressure, *SBP* systolic blood pressure, *DBP* diastolic blood pressure

**Table 5:** Characteristics of all subjects

	Grade 1	Grade 2	Grade 3	Grade 4
Number of subjects	73	73	73	73
(Male/female)	(33/40)	(37/36)	(30/43)	(31/42)
Age	40.8 ± 12.3	53.4 ± 17.6	54.1 ± 17.0	62.2 ± 14.2
Age range	25–76	25–76	25–76	25–76
Weight	62.54 ± 9.8	62.74 ± 11.53	62.30 ± 10.6	61.33 ± 10.6
Height	165.76 ± 6.7	162.13 ± 8.2	163.14 ± 7.7	164.09 ± 6.9
SBP	109.1 ± 7.3	122.9 ± 3.1	126.2 ± 6.6	153.8 ± 8.1
DBP	69.64 ± 5.6	72.4 ± 4.4	83.6 ± 4.2	88.4 ± 11.6

*SBP* systolic blood pressure, *DBP* diastolic blood pressure

All images were collected on a Ingenia 3.0T PHILIPS Medical Systems equipment with a standard head coil. The scanning parameters are as follows: repetition time (TR) = 7.90 ms, echo time (TE) = 3.50 ms, flip angle (FA) = 8°, slice thickness = 1 mm, field of view (FOV) = 250 mm and voxel dimensions 1.0 mm isotropic.

### Image process

All structural brain MR images were processed using BrainLab software [33], running automatically on Linux platform: (1) the original brain MR images were re-sampled in terms of direction, voxel size and volume according to right-hand rules. N3 bias field correction is to eliminate intensity non-uniformity [34]. (2) 3D deformable-surface-based brain extraction algorithm [35] removed non-brain tissue from the preprocessed images. (3) Level-set-based tissue segmentation algorithm [36] was used to separate GMV, WMV, CSFV, and background by limiting thickness to a biologically reasonable range with 1–6.5 mm. (4) Then, the tissue segmented images are registered to the brain atlas using a non-rigid matching algorithms derived from a concept of diffusing models [37]. The brain atlas is based on the Automated Anatomical Labeling (AAL) template with 45 labeled ROIs for each hemisphere [38]. (5) A deformable surface method accurately reconstructs inner, central, and outer cortical surfaces [39]. (6) ROI volume and cortical thickness were measured, respectively, according to the amount of voxels.

Finally, we obtained 90 cortical ROIs [40]. We computed the GMV, WMV, CSFV, Thickness, and Area for each ROI.

### Feature extraction and selection

Two types of features are used in this paper: ROI features and brain network features. The ROI features are extracted from the brain structural MR images including GMV, WMV, CSFV, Thickness and Area. Considering individual differences, the GMV, WMV, CSFV of each ROI are normalized according to the total brain volume of each subject [41], and the cortical thickness and cortical surface area of each ROI are normalized according to the standard deviation and the total cortical surface area of each subject.

Brain network features have been widely used in recent years for neuroimaging-based analysis of brain disease. The brain network features consist of Pearson correlation coefficient which are computed based on cortical thickness between ROIs. Because sub-cortical regions are not researched in this study, we neglected 12 sub-cortical ROIs of 90 cortical ROIs in the calculation [35], and finally got the 78 × 78 correlation matrix. The upper triangular elements of the matrix are used to construct the feature vector (3003-dimensional) for each subject.

Furthermore, statistical *t* test is first adopted to select the features with their *p* values less than 0.05. Then, on the basis of *t* test, mutual information method is further used to reduce feature dimensionality and improve feature representation. After the two feature selection steps, we obtained the optimal feature subsets for each feature type, respectively.

### Classification

We proposed empirical kernel mapping-based kernel extreme learning machine plus (EKM–KELM+) classifier for classification. The EKM–KELM+ algorithm has 5 parts: ROI features and brain network features, feature selection (FS), features after FS, EKM, and KELM+ classifiers. FS is used for feature reduction. EKM solves the problem of data linear indivisibility and improves the performance of classifier. KELM+ is for classification. Ensemble learning is used to get the final classification label by voting on 5 classification results. In the following parts, we will further elaborate the algorithm.

#### Empirical kernel mapping-based KELM+

**Fig. 4:**
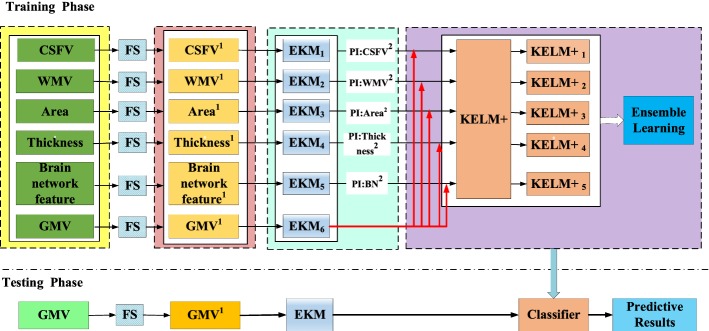
Flowchart of the proposed EKM–KELM+ algorithm. Feature selection (FS) includes t test and mutual information. In this figure, gray matter (GMV) acts as the main feature (red line), while cerebrospinal fluid (CSFV), white matter (WMV), cortical surface area (Area), and brain network features (BN, constructed by computing the Pearson correlation coefficient using mean and variance of cortical thickness between ROIs) are regard as privileged information (PI), which are help the main feature to train 5 KELM+ classifiers. Any type of feature can be treated as the main feature or PI

Figure 4 shows the flowchart of the proposed EKM–KELM+ algorithm with the following steps (GMV as the main feature as an example):

1. Six kinds of features are extracted from the brain MR images after image preprocessing, and feature selection is performed, respectively, to obtain optimal feature subsets.
2. EKM is then performed on six optimal feature subsets to generate six new enhanced feature subsets.
3. The enhanced feature subsets are then sent to KELM+ classifier. During the training stage, GMV is selected as the main feature sending to 5 KELM+ classifiers (KELM +_**1**_–KELM+_**5**_). The other five features (CSFV, WMV, Thickness, Area and brain network feature) are used as privileged information sending to KELM+1–5, respectively, which provide additional information for the main feature GMV to train 5 KELM+ classifiers.
4. The ensemble learning algorithm is finally applied to the 5 KELM+ classifiers for classification. In this work, the final classification label is decided by voting on 5 classification results.
5. During the testing stage, the GMV features extracted from structural MR images will be directly input to the 5 KELM+ classifiers (in the purple box), which then give the final classification result with the ensemble learning algorithm.

#### Empirical kernel mapping

The EKM algorithm maps original data to a given empirical feature space incrementally with explicit feature representation. Here is a brief introduction to EKM [42].

Let $\left\{ {x_{i} } \right\}_{i = 1}^{m}$ be a *d*-dimensional training samples set. The input samples space is mapped to an *r*-dimensional empirical feature space is by a particular kernel function $\varPhi^{e}$. The kernel mapping of paired $x_{i}$ and $x_{j}$ is calculated as follows:

1 $$ K_{i,j} = \varPhi_{{}}^{e} (x_{i} )^{\text{T}} \cdot \varPhi_{{}}^{e} (x_{j} ) = \ker (x_{i} ,x_{j} ), $$

where $\ker ( \cdot , \cdot )$ is a particular kernel function, leading to a kernel matrix $K = (K_{i,j} )_{m \times m}$, and $K$ is a symmetrical positive semi-definite matrix with size of $m \times m$. $K$ can be decomposed as

2 $$ K_{m \times m} = P_{m \times r} \varLambda_{r \times r} P_{r \times m}^{\text{T}} , $$

where $\varLambda$ is a diagonal matrix containing $r$ positive eigenvalues of $K$ in decreasing order, and $P$ consists of the eigenvectors corresponding to the positive eigenvalues.

The EKM to an $r$-dimension Euclidean space $\varPhi_{r}^{e}$ is then can be given as

3 $$ \varPhi_{r}^{e} (x) = \varLambda^{{{{ - 1} \mathord{\left/ {\vphantom {{ - 1} 2}} \right. \kern-0pt} 2}}} P^{\text{T}} (k(x,x_{1} ),k(x,x_{2} ), \ldots k(x,x_{m} ))^{\text{T}} . $$

Thus a sample $x$ can be mapped into empirical feature space incrementally with $\varPhi_{r}^{e} (x)$.

#### KELM

The ELM performs a classification decision by nonlinearly expanding the original features (enhancement nodes) through a single hidden layer [43].

In ELM, the output weight *β* can be calculated by ridge regression as

4 $$ \beta { = }\left( {{\text{H}}^{\text{T}} H + I/C} \right)^{ - 1} H^{\text{T}} T, $$

where *T* is a label matrix, *C* is the regularization parameter, which represents the trade-off between the minimization of training errors and the maximization of the marginal distance and *H* is the enhanced matrix.

To overcome the problem of randomness in ELM, the kernel trick is then introduced into ELM as shown in Fig. 4. For KELM [23], we define the kernel matrices as

5 $$ \tilde{\varOmega } = HH^{\text{T}} :\tilde{\varOmega }_{i,j} = \tilde{K}(x_{i} ,x_{j} ),\quad i,j = 1,2 \ldots n, $$

where *K* is a linear kernel function and $\tilde{K}$ represents a nonlinear kernel function.

The output of KELM is then given by

6 $$ f(x) = \left( {\left[ {\begin{array}{*{20}c} {K(x,x_{1} )} \\ \vdots \\ {K(x,x_{n} )} \\ \end{array} } \right]} \right) \times \left( {\frac{1}{C} + \tilde{\varOmega }} \right)^{ - 1} T, $$

with the output weights calculated by the ridge regression as

7 $$ \beta { = }\left( {\frac{1}{C} + \tilde{\varOmega }} \right)^{ - 1} T. $$

#### KELM+

ELM+ successfully integrates the LUPI paradigm to ELM, which has simpler optimization constraint than the commonly used SVM+.

Define a set of training data $\left\{ {\left( {x_{i} ,P{}_{i},t_{i} } \right)} \right.|x_{i} \in R^{{d_{1} }} ,P_{i} \in R^{{d_{2} }} ,t_{i} \in R^{m} ,i = 1 \ldots n\}$, where $\{ P_{i} \in R^{{d_{2} }} ,i = 1 \ldots n\}$ is a set of PI. In LUPI paradigm, ELM+ is formulated as

$$ \min_{{\beta ,\tilde{\beta }}} L_{{{\text{ELM}} + }} = \frac{1}{2}\left\| \beta \right\|^{2} + \frac{\varepsilon }{2}||\tilde{\beta }||^{2} + \frac{C}{2}\sum\limits_{k = 1}^{n} {\left( {\tilde{h}\left( {P_{k} } \right)\tilde{\beta }} \right)^{2} } , $$

8 $$ {\text{s}} . {\text{t}} . { }\;\;\;\;h(x_{k} )\beta = t_{k} - \tilde{h}\left( {P_{k} } \right)\tilde{\beta },\forall 1 \le k \le n, $$

where *ɛ* is a regularization coefficient, $h(x_{i} )$ and $\tilde{h}(P_{i} )$ are concatenated vector, and $\tilde{\beta }$ is an output weight vector in the privileged feature space.

The Lagrangian function is then constructed to solve the optimization problem in Eq. (8) by

9 $$ L_{{{\text{ELM}} + }} = \frac{1}{2}\left\| \beta \right\|^{2} + \frac{\varepsilon }{2}\left\| {\tilde{\beta }} \right\|^{2} + C\sum\limits_{k = 1}^{n} {\tilde{h}\left( {P_{k} } \right)\tilde{\beta }} - \sum\limits_{k = 1}^{n} {\left( {h\left( {x_{k} } \right)\beta - t_{k} + \tilde{h}\left( {P_{k} } \right)\tilde{\beta }} \right)} , $$

where $\lambda = \left[ {\lambda_{1} , \ldots ,\lambda_{n} } \right]^{\text{T}}$ are Lagrange multipliers.

After using the Karush–Kuhn–Tucker (KKT) condition to calculate the saddle points of the Lagrangian function, we have

10 $$ \beta = H^{\text{T}} \lambda , $$

11 $$ \tilde{\beta } = \frac{1}{\varepsilon }\left( {\tilde{H}^{\text{T}} \lambda - \tilde{H}^{\text{T}} C1} \right), $$

12 $$ \tilde{h}(p_{i} )\tilde{\beta } - t_{i} = 0 \quad \forall 1 \le i \le n. $$

By substituting Eqs. (10) and (11) into (12), we have

13 $$ \left( {\frac{1}{\varepsilon }\tilde{H}\tilde{H}^{\text{T}} } \right)\lambda = T - \frac{C1}{\varepsilon }\tilde{H}\tilde{H}^{\text{T}} . $$

After combining Eqs. (10) and (13), the closed-form solution to the ELM+ is given by

14 $$ \beta {\text{ = H}}^{T} \left( {\frac{1}{\varepsilon }\tilde{H}\tilde{H}^{\text{T}} } \right)^{ - 1} \left( {T - \frac{C1}{\varepsilon }\tilde{H}\tilde{H}^{\text{T}} } \right). $$

Moreover, $\frac{1}{C}$ is added to Eq. (13) so as to avoid singularity and guarantee the stability for ELM+, which leads to the following closed-form solution:

15 $$ \beta = H^{\text{T}} \left( {\frac{1}{\varepsilon }\tilde{H}\tilde{H}^{\text{T}} + \frac{1}{C}} \right)\left( {T - \frac{C1}{\varepsilon }\tilde{H}\tilde{H}^{\text{T}} } \right). $$

The output function of the ELM+ is defined as

16 $$ f(x) = h(x)\beta = h(x)H^{\text{T}} \left( {\frac{1}{\varepsilon }\tilde{H}\tilde{H}^{\text{T}} + \frac{1}{C}} \right)^{ - 1} \left( {T - \frac{C1}{\varepsilon }\tilde{H}\tilde{H}^{\text{T}} } \right). $$

Although ELM+ can implement the LUPI-based classification task, it also suffers from the same problem of randomness as ELM. Therefore, the kernel-based ELM+ algorithm is then proposed.

For the KELM+, we define the kernel matrices with same structure as Eqs. (4) and (5), the output weight vector is then given by

17 $$ \beta_{\text{kernel}} = \left( {\frac{1}{\varepsilon }\tilde{\varOmega } + \frac{1}{C}} \right)^{ - 1} \left( {T - \frac{C1}{\varepsilon }\tilde{\varOmega }} \right). $$

The output of KELM+ is finally calculated as

18 $$ f_{\text{kernel}} (x) = \left( {\left[ {\begin{array}{*{20}c} {K(x,x_{1} )} \\ \vdots \\ {K(x,x_{n} )} \\ \end{array} } \right]} \right) \times \left( {\frac{1}{\varepsilon }\tilde{\varOmega } + \frac{1}{C}} \right)^{ - 1} \left( {T - \frac{C1}{\varepsilon }\tilde{\varOmega }} \right). $$

For multiclass cases, the predicted class label of a testing point is the index number of the output node, which has the highest output value for the given testing samples

19 $$ {\text{label}}(x) = \mathop {\arg \text{max} \quad f_{j} (x)}\limits_{{j \in \left\{ {1, \ldots ,m} \right\}}} . $$

## Data Availability

The datasets used and/or analyzed during the current study are available from the corresponding author on reasonable request.

## Abbreviations

MRI: magnetic resonance imaging
EKM: empirical kernel mapping
KELM+: kernel extreme learning machine plus
ROI: regions of interest
ACC/AHA: American College of Cardiology/American Heart Association
BP: blood pressure
GMV: gray matter volume
WMV: white matter volume
CSFV: cerebrospinal fluid volume
Thickness: cortical thickness
Area: cortical surface area
LUPI: learning using privileged information
PI: privileged information
